# The Institutional Worker

**Published:** 1920-10-16

**Authors:** 


### INSTITUTIONAL WORKER

**Being a Special Supplement to "The Hospital"**

### OUR BUREAU OF INFORMATION.

### Rules for Correspondents

1. Every letter must be accompanied by the coupon to be cut from the back cover (inside page) of The Hospital, current issue, and must contain the name and address of the correspondent with pseudonym for publication if desired. Replies by post cannot be given save under exceptional circumstances at the Editor's discretion.

2. Letters from Approved Homes in reply to special needs published in the Bureau should state terms and full particulars, and be sent prepaid under cover to the Editor of the Bureau with name written across coupon for identification.

3. Proprietors of Homes which have not yet been entered on the List of Approved Homes, but have spare accommodation likely to suit special needs, are invited to write for an application form for registration. The fee for registration, which includes two announcements of the Home in the Bureau and other privileges, is 10s.

4. All communications to be addressed to the Editor of The Hospital, 28 Southampton Street, Strand. London, W.C. 2, and marked "Bureau of Information."

### INSTITUTION FACTS AND FIGURES.

### The Question Box.

#### THE HOUSEKEEPER'S STORE CUPBOARD.

The question was :

**State how and to what extent the store cupboard has been replenished by produce from the hospital garden, the amount of jam, preserves, &c., so supplied, the cost, and whether any extra service was obtained to gather or preserve the fruit, vegetables, &c.**

By "Wales."

Fruit Preserving.

The cost of fruit has been too high to go in for preserving on a large scale this year, and the crop of fruit was so poor that it was not possible to get any for preserving from our own garden except rhubarb; this also was scarce, but 60 lb. of jam was made. Two cwt. of sugar was ordered for preserving, according to the control regulations. As the jam was made for immediate use, an allowance of 10 oz. of sugar to the pound was given instead of 12 oz., which would have been required had the jam been for keeping longer.

Yellow plums were bought a few weeks ago, £2 6s. being paid for 144 lb.; taking them thus in bulk they worked out cheaper, but 20 lb. were too bruised for preserving. Out of the remainder 120 lb. of jam was made. Half-a-pound of sugar to 1 lb. of plums was used, as the plums were sweet and the jam not intended for keeping.

When the garden produce is all gathered we hope to make from our own produce vegetable marrow jam, green tomato jam, carrot jam, and some cucumber marmalade.

Vegetables Stored and Preserved.

This year we have pickled small onions, cauliflower, red cabbage, green tomato. In addition we have made a certain quantity of chutney and also mixed pickles (onions, cucumber, cauliflower, and French beans).

Vegetable marrows (fourteen) have been hung for winter use. One dozen bottles of green peas and a large jar containing 40 lb. of French beans have up to the present been preserved.

Parsley, mint, thyme, marjoram have been dried and hung in bags. A certain quantity of mint has been preserved in vinegar and made airtight; this keeps green for mint sauce. In addition we have a large quantity of beetroot: these are stored with the turnips, carrots, potatoes, etc., and the beetroot is boiled for use as required.

We hope to have stored sufficient potatoes to last until next season, and sufficient other vegetables, including onions, to keep us going until after Christmas.

Preserved Eggs.—These were bought for 3¼d. each in the early summer, and 400 put down in waterglass.

It is impossible at present to estimate the exact quantity of preserve made, etc., as a good deal of produce is still in the ground and the tomatoes are yet ripening. No additional help was required to gather the produce; the gardener, with the help of the boy, did the work. I may add that all produce has to be paid for; all vegetables, etc., are checked by the gardener, and the account presented every month, along with the other bills, and market prices quoted—thus a check is kept on the produce and a means of knowing whether the garden pays or not.

The cook, under matron's supervision, did the pickling and preserving.

### Co-operative Buying.

While it is recognised that the co-operative system among the hospitals for the purchase of all requisites might be made of immense value in the reduction of expenditure and labour, no attempt on any considerable scale to establish such a system has been successful. Messrs. H. Wright and Co., of 44-46 Pear Tree Street, London, E.C. 1, merchants and import and export agents, who claim to have had a large experience as contractors to many hospitals, are now putting forward a proposal to purchase for the hospitals direct from the manufacturers requisites of every description required. Providing they can get sufficient support from the hospitals, they will be willing to deal with orders on the following basis :—To receive orders either by 'phone or post (in the former case to be confirmed in writing the same day); to place them immediately with firms actually manufacturing the articles required, and in every instance choosing the best and cheapest market; to charge for them at net manufacturers' prices; to send copies of manufacturers' invoices with the goods for checking purposes, and subsequently to send the manufacturers' actual invoices bearing the order number. For these services Messrs. Wright and Co. propose to charge a commission of 5 per cent. Payment would be made to them in accordance with invoices delivered, plus 5 per cent. Thus each month the hospital would liquidate its obligations by drawing one cheque instead of nearly one hundred. It is proposed to provide for urgent and special requirements in London by special delivery van. Country orders would be sent to the nearest large railway terminus and personally despatched on the first available passenger train. There are certain obvious advantages in this suggested plan. It remains to be seen, however, whether the organisation can be so perfected as to avoid delays in transport. The offer is well worth careful consideration and an initial trial to a limited extent.

### Question for October.

### PATIENTS' WAITING-LISTS.

**Describe a simple method of recording names of patients awaiting admission, showing at a glance their proper turn for admission having regard both to period of waiting and the medical urgency of their respective cases.**

**A minimum payment of 10s. 6d. will be made for each published answer.)**

RULES.

The following rules must be observed:—

1. Contributions must be written on one side of the paper. Conciseness and terseness are desirable features. The MS. must bear the name and address of the sender and be accompanied by coupon to be cut from the back cover (inside page) of the current issue of The Hospital. A pseudonym must be chosen if the name is not to be published.

2. Contributions must be addressed to the Editor of The Hospital Institutional Worker Supplement, 28 & 29 Southampton Street, Strand, London, W.C. 2, should reach him before the end of the current month, and be marked in the left-hand corner "Facts and Figures."

### QUESTIONS INVITED.

In connection with our Question Box, we offer every month five shillings each for the two best questions which are sent in for consideration. The questions may be on any institutional subject and concern any department, from the secretary's to the porter's, or the matron's to the domestic staff; the one essential is that they must be practical and deal with points that have a definite relation to hospital or institutional administration, upkeep, finance, or management. Points relating to artificers' work, housekeeping, the laundry, out-patients, and other practical matters will be welcome. It is hoped that thus, when difficult or doubtful points arise in the course of their work, institutional workers will be encouraged to put them in the form of a question and send them to the Editor, The Hospital Bureau of Information, 28 Southampton Street, Strand, W.C. 2, marked "Question Box."

The best questions will be published in due course and our readers will be given the opportunity of answering them. Thus all institutional workers may be able to co-operate with the view of helping the work and smoothing the difficulties of each other. In short, we want the Question Box of the *Institutional Worker* to be freely used by every worker habitually.

**The Editor will be glad to receive correspondence and to consider contributions upon all subjects relating to institutional work which affect the welfare of institutional workers.**

### ENQUIRIES AND ANSWERS.

### *THE SICK AND IN NEED.*

### Accommodation for Nurse in Newcastle.

We suggest that you write to the Secretary of the Residential Club for Nurses, Windsor Crescent, Newcastle-on-Tyne, which has just been opened under the auspices of the Newcastle Centre of the College of Nursing. An inclusive weekly charge is made for bed and breakfast only, 25s., or two guineas for full board.—Nurse A.

### *EMPLOYMENT AND TRAINING.*

### Position of Matron.

We are afraid that the only suggestion we can make to enable you to obtain the position you require is that you reply to advertisements which appear from week to week in the nursing journals. Your experience ought eventually to meet with the success you desire. A coupon should be enclosed with each inquiry; we cannot give postal replies.—H. P.

### Evening and Week-end Work.

It might be possible for you to obtain evening work from a medical man in the district in which you are working. Make your needs known by advertisement.—C. W. H.

### Prison Nursing.

The address of the Penal Reform League is 68a Park Hill Road, N.W. 3. Write to the Secretary of the League, and he will no doubt give you particulars of prison nursing. A coupon should be enclosed with each inquiry. We regret we cannot give postal replies.—Sympathetic.

### Training for Female Sanitary Inspector in Ireland.

Yes, it is, we understand, possible to train as a female sanitary inspector in Ireland, either Dublin or Belfast. Your best plan would be to write to the Secretary, Royal Sanitary Institute, Buckingham Palace Road, London, S.W. 1, and ask for full particulars of the training. Enclose stamped envelope for reply.—Colleen.

### Chartered Society of Massage and Medical Gymnastics.

You are eligible for registered membership of the Chartered Society of Massage and Medical Gymnastics without further examination. In accordance with by-law 37, members of the I.S.T.M. and the I.M.R.G. are put on the register upon payment of a fee of two guineas.—"Masseuse."

### Book of Value to Matron.

Obtain "Training School Methods for Institutional Nurses," by Charlotte A. Aikens. The price is 10s. 6d. net. The publishers are W. B. Saunders & Co., but a good newsagent would obtain it for you. This book is of great value to any holding responsible positions in the hospital world.—"Kew."

### *MISCELLANEOUS.*

### Address Wanted.

The address of the Welfare Workers' Institute is 11 Adam Street, Adelphi, London, W.C.—Pip.

### Repairs of Electric Lamps.

Provided that you have kept your spent electric lamps intact you can get them repaired at a price considerably less than a new lamp. The Allies Electric Lamp Repairing Co., Ltd., of Montgomery Street, Hammersmith, W., undertake this work. The process adopted is to cut the bulb an inch or two below the cap, replace the filament, weld together the parts of the bulb, and re-exhaust. The remade lamp is guaranteed by the Repairing Company, and will be replaced if found defective. The price per lamp ranges from 1s. 9d. to 2s., in accordance with the voltage. A discount of 10 per cent. is allowed on a quantity not less than 100. For larger quantities special quotions are given. The price of a new lamp (net) ranges from 2s. 3d. to 2s. 6d.—Economy.

### New Examining and Operating Table.

Probably one of the most perfect types of operating tables is that provided by M. Schaerer, of Berne (London address, 41 Berners Street, W.1). Simple and easy of manipulation, it provides for every possible position required by the surgeon. The table is mounted on a heavy tripod, and is raised or lowered over a distance of 12 inches by means of an oil pump. In the lowest position the table top is about 33 inches from the floor, in the highest about 45 inches. This model would repay examination, and though the price (£180, excluding additional accessories) is high, on the other hand the construction and workmanship are of the highest order.—Medico.

### Electrically-heated Bandages.

Messrs. M. E. Du Bois and Co. are the manufacturers of the electrically heated bandage to which you refer. This is one of many electrical devices made by this company.—Enquirer.

### X-Ray Films.

Films are undoubtedly cheaper than plates for *x*-ray work, and offer a great advantage over the latter when the question of storage and transmission from place to place are taken into consideration. Regarding the excellence of the resultant negative from the Kodak film there is no question. If you are interested, we suggest that you visit the annual exhibition of the Royal Photographic Society, 35 Russell Square, W.C. 1. Demonstrations on the subject are given on Tuesdays and Thursdays from 4 to 6 p.m. The exhibition lasts until October 30.—Radiographer.

